# Single Cell Transcriptomic Analysis Reveals Organ Specific Pericyte Markers and Identities

**DOI:** 10.3389/fcvm.2022.876591

**Published:** 2022-06-01

**Authors:** Seung-Han Baek, Enrico Maiorino, Hyunbum Kim, Kimberly Glass, Benjamin A. Raby, Ke Yuan

**Affiliations:** ^1^Division of Pulmonary Medicine, Department of Pediatrics, Boston Children's Hospital and Harvard Medical School, Boston, MA, United States; ^2^Channing Division of Network Medicine, Brigham and Women's Hospital and Harvard Medical School, Boston, MA, United States

**Keywords:** pericytes, single cell RNA sequencing, heart, lung, tissue-specific

## Abstract

Pericytes are mesenchymal-derived mural cells that wrap around capillaries and directly contact endothelial cells. Present throughout the body, including the cardiovascular system, pericytes are proposed to have multipotent cell-like properties and are involved in numerous biological processes, including regulation of vascular development, maturation, permeability, and homeostasis. Despite their physiological importance, the functional heterogeneity, differentiation process, and pathological roles of pericytes are not yet clearly understood, in part due to the inability to reliably distinguish them from other mural cell populations. Our study focused on identifying pericyte-specific markers by analyzing single-cell RNA sequencing data from tissue-specific mouse pericyte populations generated by the Tabula Muris Senis. We identified the mural cell cluster in murine lung, heart, kidney, and bladder that expressed either of two known pericyte markers, *Cspg4* or *Pdgfrb*. We further defined pericytes as those cells that co-expressed both markers within this cluster. Single-cell differential expression gene analysis compared this subset with other clusters that identified potential pericyte marker candidates, including *Kcnk3* (in the lung); *Rgs4* (in the heart); *Myh11* and *Kcna5* (in the kidney); *Pcp4l1* (in the bladder); and *Higd1b* (in lung and heart). In addition, we identified novel markers of tissue-specific pericytes and signaling pathways that may be involved in maintaining their identity. Moreover, the identified markers were further validated in Human Lung Cell Atlas and human heart single-cell RNAseq databases. Intriguingly, we found that markers of heart and lung pericytes in mice were conserved in human heart and lung pericytes. In this study, we, for the first time, identified specific pericyte markers among lung, heart, kidney, and bladder and reveal differentially expressed genes and functional relationships between mural cells.

## Introduction

Pericytes are mesenchymal-derived mural cells that cover capillary networks throughout the circulatory system. Partially surrounding capillary endothelial cells of precapillary arterioles and extending to post-capillary venules, pericytes are indispensable to the function of all organs by providing capillary structural support and facilitating capillary and endothelial cell homeostasis and metabolism, contributing to vasoregulation of blood flow, capillary basement membrane synthesis, and initiation of angiogenesis (1–4). Pericytes are suggested to have multipotent cell-like properties. This plasticity has been implicated in the pathogenesis of many cardiovascular, pulmonary, and central nervous systems disorders and the metastasis and angiogenesis of diverse cancers (5–9).

Despite their physiological importance, a significant challenge in studying pericytes is the lack of a consensus definition to distinguish pericytes from other mural cell populations. Pericytes are recognized by their distinct morphology, consisting of an oval cell body and long extending processes that circumscribe vascular structures. Two proteins have been traditionally employed as pericyte markers: Chondroitin Sulfate Proteoglycan 4 (*Cspg4*) (also known as neural/glial antigen 2, NG2) and Platelet-Derived Growth Factor Receptor b (*Pdgfrb*), a cell surface tyrosine kinase receptor for members of the platelet-derived growth factor family (10, 11). However, these markers have generally failed to serve as unique pericyte markers. They are also expressed in other mesenchymal cell types, including smooth muscle (*Acta2* and *Tagln*) and fibroblasts (*Pdgfra, Lum*, and *Dcn*) (12, 13). Adding to this ambiguity, *Pdgfrb*+ or *Cspg4*+ cells expressing smooth muscle markers are inconsistently classified as either non-pericyte cells or a specific pericyte subset (e.g., *Acta2-*high expressing *Pdgfrb*+ pericytes in neonatal mouse lung) (14). Recent advances in single-cell RNA sequencing (scRNA-seq) have demonstrated substantial between-organ differences in pericytes gene expression, likely reflecting fundamental organ-specific differences in pericyte function. For example, compared with lung-derived pericytes, transcripts expressed in pericytes from the brain were enriched for genes associated with transmembrane transporter activity, consistent with the known role of pericytes in the blood-brain barrier function (15). Whether such heterogeneity in cellular phenotype and function extends to the vascular beds of other tissues remains largely unknown. The identification of unique tissue-specific pericyte markers has the potential to improve our understanding of pericyte diversity between and within the tissue and their impact on health and disease.

This study identified tissue-specific pericyte markers by analyzing murine and human scRNA-seq datasets generated in multiple tissues, including the lung, heart, kidney, and bladder. Using stringent criteria for defining pericyte clusters (*Cspg4*+*/Pdgfrb*+ dual positive), we identified several candidate markers whose expression was restricted to these “stringent pericytes,” several of which exhibited tissue-specific properties in both mice and humans. We demonstrated the ability of a subset to distinguish between lung and heart-derived human pericytes, illustrating their potential use in future studies.

## Results

### Identification of Pericyte Clusters From Mouse Lung, Heart, Kidney, and Bladder

**Figure 1:**
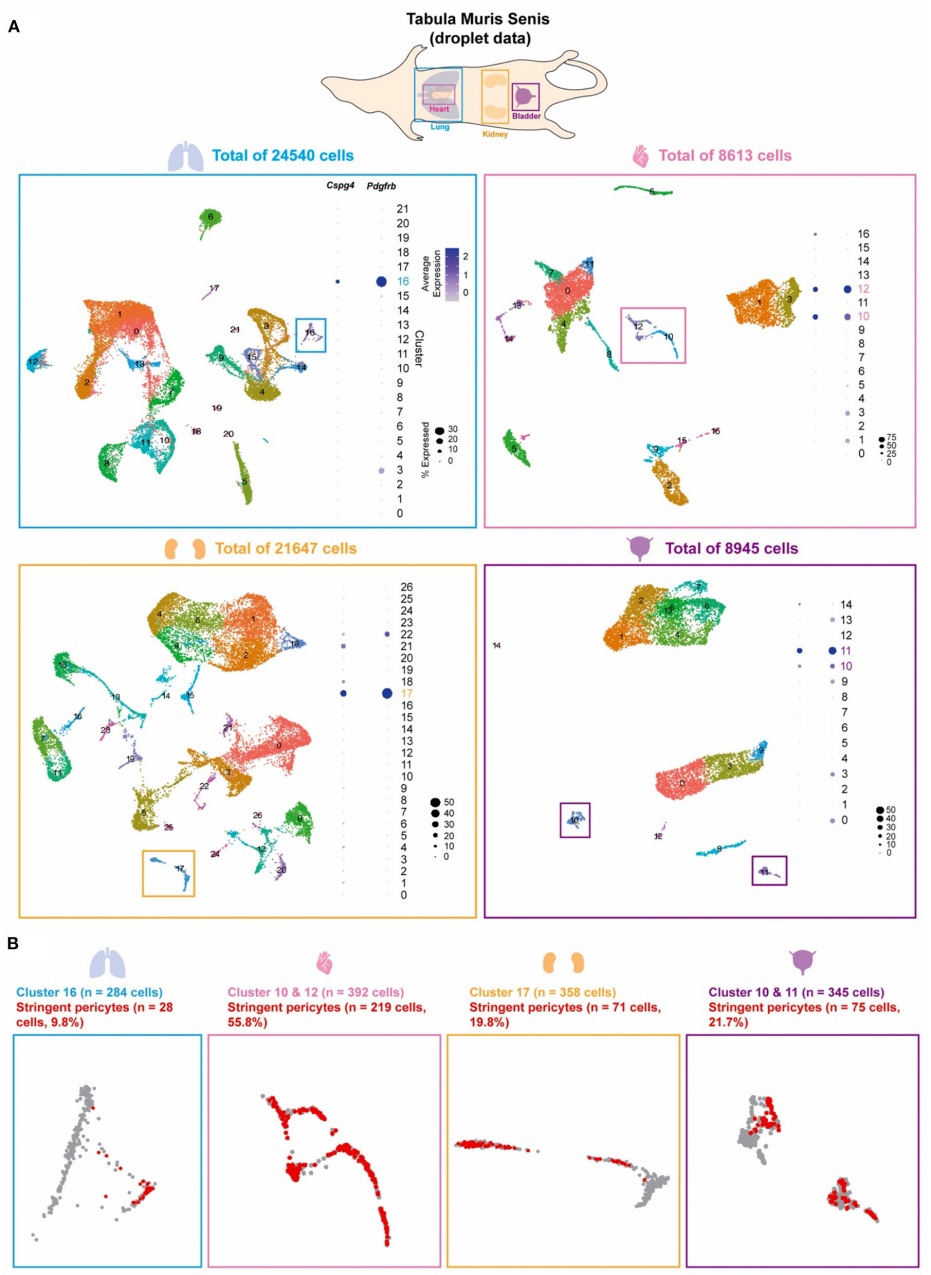
Pericyte clusters are identified from mouse lung, heart, kidney, and bladder. **(A)** The Tabula Muris Senis dataset of the lung, heart, kidney, and bladder were used to identify pericytes within the cell population of each tissue. Graph-based unsupervised clustering identified 22, 17, 27, and 15 clusters for the lung, heart, kidney, and bladder tissue, respectively. **(B)** Pericyte-enriched clusters for each tissue were identified based on the expression of previously accepted pericyte markers *Cspg4* and *Pdgfrb*. Cells that co-expressed Cspg4 and Pdgfrb within the identified pericyte-enriched clusters were stringent pericytes (shown in red).

The Tabula Muris Senis compendium is a single-cell survey across 23 C57BL/6JN mice tissues. From its website (https://tabula-muris-senis.ds.czbiohub.org/all/droplet/), one of the two previously accepted pericyte markers, *Cspg4* or *Pdgfrb*, was positively expressed mostly in lung, heart, kidney, and bladder (Supplementary Figure 1). We therefore moved forward to analyze scRNA-seq data from lung (*n* = 16 mice; 24,540 cells), heart (*n* = 11; 8,613), kidney (*n* = 16; 21,647), and bladder (*n* = 8; 8,945) obtained from the Senis (Figure 1A) (16). Unsupervised clustering revealed substantial cellular heterogeneity within each tissue, with the number of distinct cell clusters ranging from 15 in the bladder to 27 in the kidney. To identify pericyte-enriched clusters, we first considered the individual and joint expression of two previously accepted pericyte markers, *Cspg4* and *Pdgfrb*. Consistent with prior observations (10, 11, 14), the preponderance of cells expressing either *Cspg4* and *Pdgfrb* localized to a limited number of clusters (one each in lung and kidney, two in heart and bladder). In contrast to all other clusters, which either consisted of few *Cspg4* or *Pdgfrb*-expressing cells or had low/undetectable expression of both markers, these clusters were tentatively deemed pericyte-enriched because of their large portions of cells expressing high levels of both *Cspg4* and *Pdgfrb* (Figure 1A). However, within these pericyte-enriched clusters, substantial heterogeneity in the expression of *Cspg4* and *Pdgfrb* was observed. Therefore, within these pericyte-enriched clusters, we defined a set of “stringent pericytes” by considering only those cells that expressed both *Cspg4* and *Pdgfrb* (Figure 1B). A total of 393 cells met these criteria (28.4% of all cells), including 28 in the lung, 219 in the heart, 71 in the kidney, and 75 in the bladder, with the proportion of stringent pericytes within each cluster varying widely by tissue (highest in heart, 55.8%; lowest in the lung, 9.8%). These refined subsets were considered tissue-specific stringent pericytes in all downstream analyses.

### Identification of Tissue-Specific Pericyte Markers

**Figure 2:**
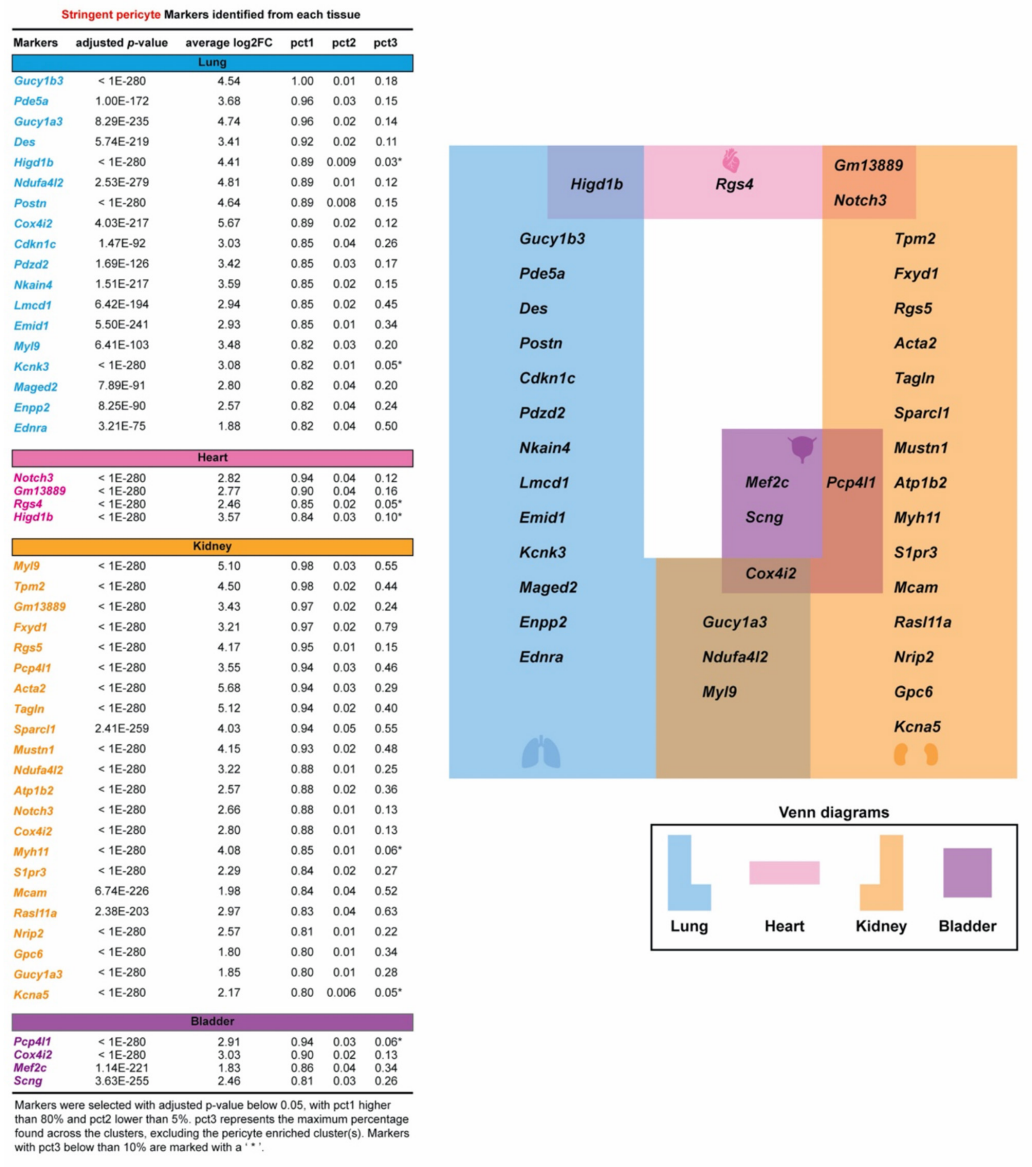
Specific pericyte markers in four organs are identified. Potential pericyte markers for each tissue. Potential pericyte markers were selected based on the differential expression (DE) analysis using the Wilcoxon rank sum test to compare the expression levels of transcripts in the tissue-specific stringent pericytes vs. all other cells within the corresponding tissue. Pericyte-enriched transcripts with an adjusted *p*-value below 0.05 were selected. The subset of these transcripts detected in more than 80% of the stringent pericytes and in <5% in all other cells were then chosen as potential pericyte markers for each tissue. pct3 represents the maximum percentage found across the clusters, excluding the pericyte-enriched cluster(s). Markers with pct3 below 10% are shown with an asterisk (*).

We conducted a differential expression (DE) analysis between the stringent pericytes and all other cells based on the Wilcoxon rank-sum test to identify potential pericyte-specific markers. We identified transcripts that were enriched in the stringent pericytes in each tissue, prioritizing transcripts with high pericyte DE (adjusted *p*-value < 0.05) that were expressed (count > 0) in at least 80% of the stringent pericytes but <5% of all other cells. Based on these criteria, we identified 18, 4, 22, and 4 potential pericyte markers for the lung, heart, kidney, and bladder, respectively (Figure 2, left). No marker fulfilled these criteria in all four cell types. However, several markers were common to two or three tissues (Figure 2, right), including *Cox4i2* (in lung, kidney, and bladder); *Gucy1a3, Ndufa4i2*, and *Myl9* (in lung and kidney); *Gm13889* and *Notch3* (in heart and kidney); *Pcp4l1* (in kidney and bladder); and *Higd1b* (in lung and heart). Also, for each cluster, we determined the percentage of cells expressing the identified markers (Figure 2, left and Supplementary Figure 6). The pct3 represents the maximum percentage found across the clusters, excluding the pericyte enriched cluster(s). When considering markers with pct3 below 10%, we identified *Kcnk3* (in the lung); *Rgs4* (in the heart); *Myh11* and *Kcna5* (in the kidney); *Pcp4l1* (in the bladder); and *Higd1b* (in lung and heart), which showed that they were very specific to pericyte-enriched clusters.

### Pathway Enrichment Analysis of Pericytes in Each Tissue

**Figure 3:**
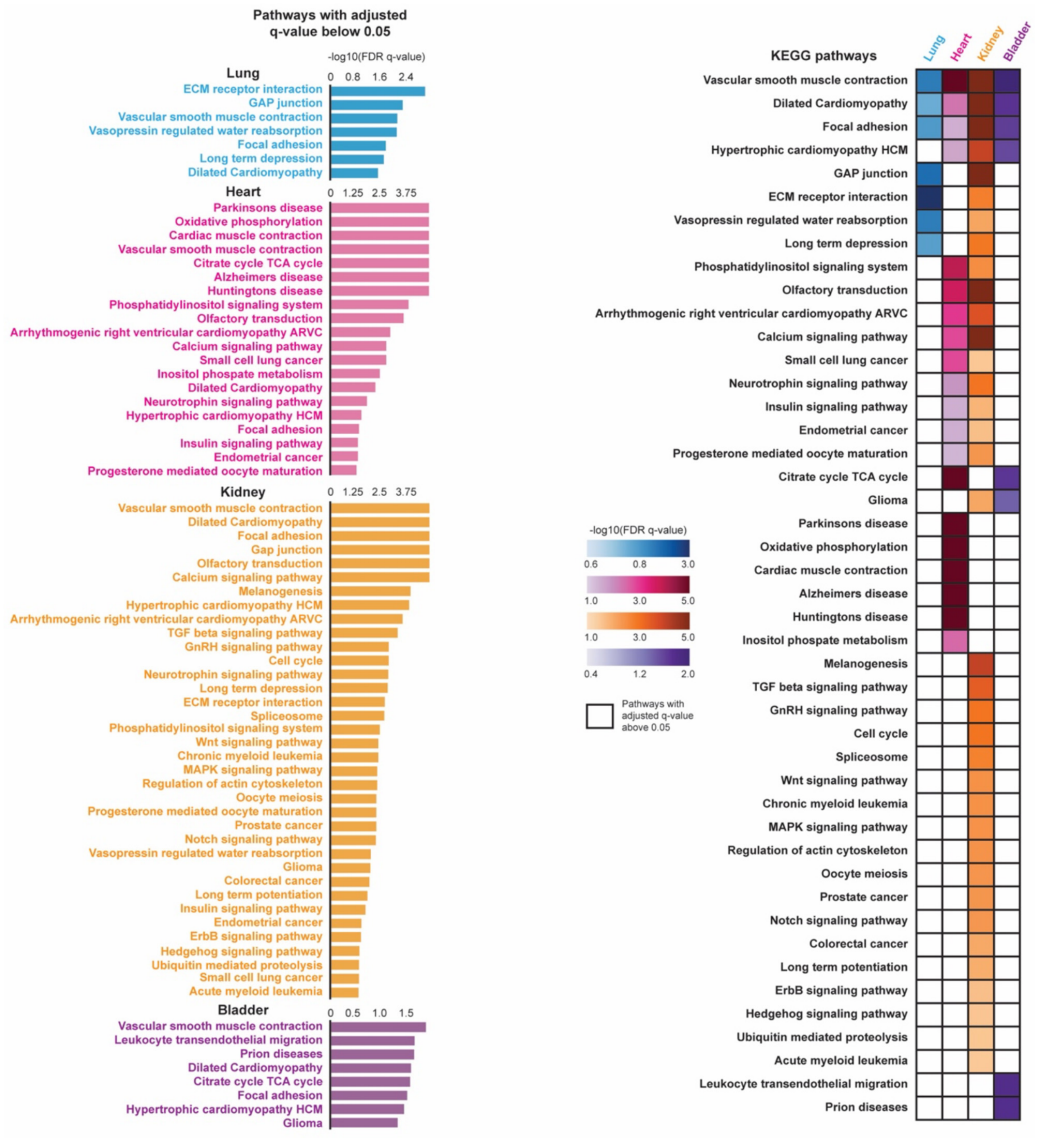
Pathway enrichment analysis of pericytes in four organs are shown. Pre-ranked gene set enrichment analysis (GSEA) with the Kyoto Encyclopedia of Genes and Genomes (KEGG) pathway database was conducted on the DE genes between the tissue-specific stringent pericytes and all other cells of its corresponding tissue. The DE genes were sorted based on their average log2(Fold Change) in a descending order to generate the pre-ranked gene list. Enriched pathways were identified for each tissue-specific stringent pericytes based on their adjusted q-value (*q* < 0.05). The identified enriched pathways of the tissue-specific stringent pericytes were compared across four tissue types.

To determine pathways associated with stringent pericytes across the lung, heart, kidney, and bladder, we conducted a pre-ranked Gene Set Enrichment Analysis (GSEA) (17) (Figure 3). First, genes were sorted based on their average log2(Fold Change) when comparing their expression in tissue-specific stringent pericytes to all cells in the corresponding tissue. GSEA was then performed on the resulting pre-ranked list using the Kyoto Encyclopedia of Genes and Genomes pathway database (18). We identified 7, 20, 36, and 8 pathways enriched (adjusted *q*-value < 0.05) in the stringent pericytes of the lung, heart, kidney, and bladder, respectively.

Vascular smooth muscle contraction dilated cardiomyopathy, and focal adhesion were top enriched pathways in the stringent pericytes of all four tissues, suggesting pathways involved in pericyte physiological and metabolic activity (Figure 3). This may be because pericytes share a similar lineage with smooth muscle cells (SMCs) and differentiate into SMCs under stress. Additionally, as broadly defined mural cells, their biological function can be identical to vital regulators of vessel structure support, permeability, and homeostasis (19, 20).

The other identified pathways in heart tissue were involved with neurodegenerative disorders and cardiac muscle contraction. Pericytes are one of the key regulators of the blood-brain barrier. Their involvement in the pathogenesis of neurological disorders may be associated with cardiovascular manifestations under the heart-brain axis (7, 21, 22). Pathways related to cancers are also enriched in the kidney-specific stringent pericytes. Pericytes contribute to the invasion and metastasis of cancer or tissue fibrosis through pericyte-fibroblast transition (PFT) mechanisms, which may be highlighted by the cell cycle, TGFb/Wnt/MAPK/Notch/Hedgehog pathways (19, 23). The urinary bladder is highly enriched with blood vessels, though the function of pericytes in this organ has been sorely under-investigated.

### Expression of Pericyte Markers in Human Lung and Heart

**Figure 4:**
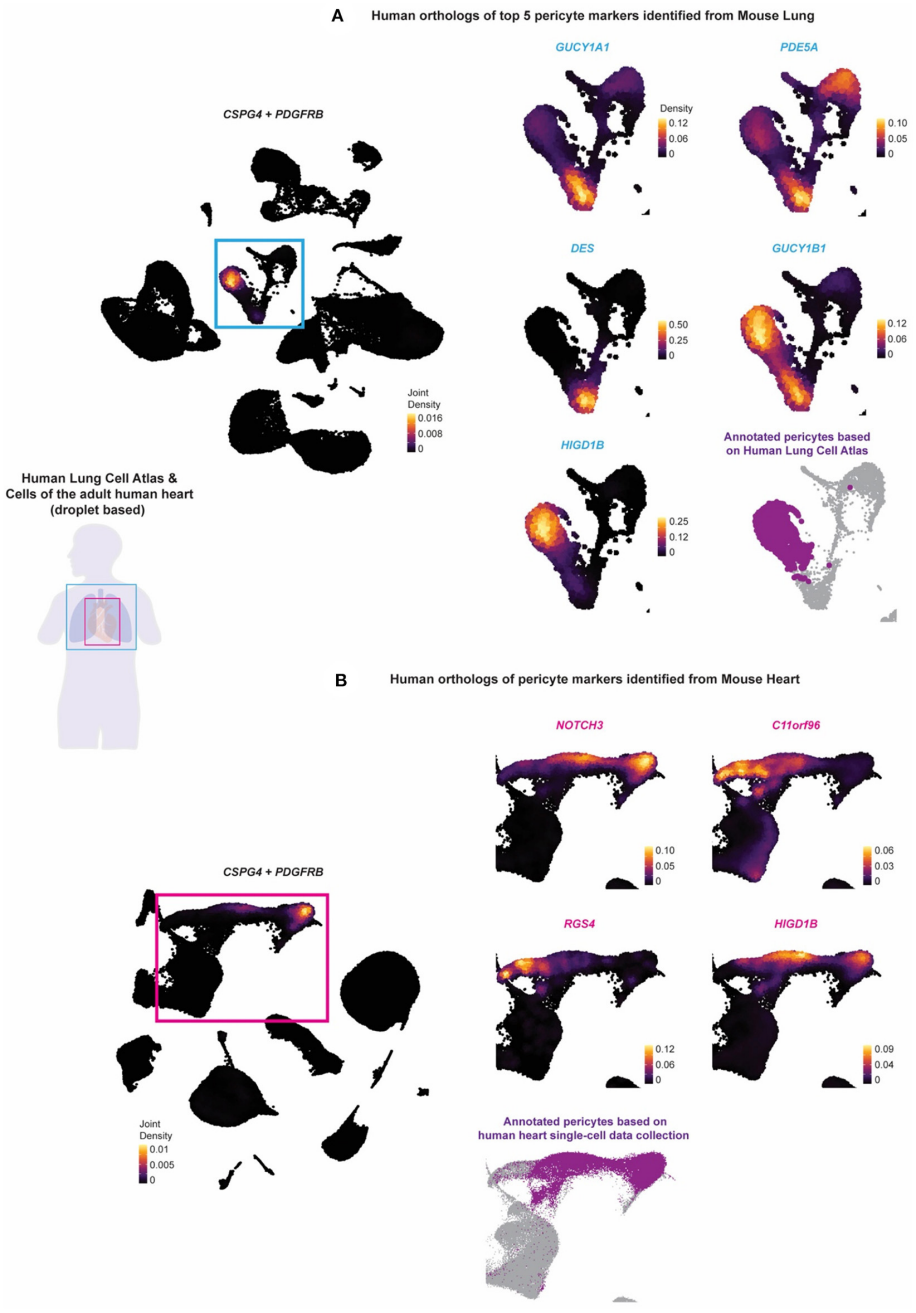
The identified murine lung and pericyte markers are tested on human lung and heart scRNAseq data. The expression of human orthologs of the potential pericyte markers identified from the stringent pericytes of the mouse lung and heart tissue were tested against the **(A)** Human Lung Cell Atlas and the **(B)** collection of the cells of the adult human heart to validate whether they can be used to annotate pericytes in the human lung and heart tissue. Purple (fond color) indicated the annotated pericytes of the two human datasets. The Human Lung Cell Atlas annotated pericytes based on *CSPG4, PDGFRB*, and *TRPC6*, while the human heart pericytes were annotated based on *RGS5, ABCC9*, and *KCNJ8*. Light blue (fond color) genes indicated the human orthologs of the top 5 potential pericyte markers identified from the mouse lung. Pink (fond color) genes indicated the human orthologs of all the potential pericyte markers identified from the mouse heart.

To determine whether the potential pericyte markers identified from the mouse tissue were conserved in humans, we analyzed single-cell data from the Human Lung Cell Atlas and adult human heart single-cell data collected by Litvinukova et al. (24, 25) (Figure 4). The Human Lung Cell Atlas data consisted of scRNA-seq on 75,000 human lung cells and circulating blood collected from three individuals aged 75 (male), 46 (male), and 51 years (female) (24). The human heart data consisted of scRNA-seq on 486,134 human cells from all heart compartments and collected 14 individuals of both sexes with ages ranging from 40 to 75 years (25). To unify our analysis of these data with the Tabula Muris Senis data analysis, we selected only droplet-based scRNA-seq data processed using the 10 × Genomics platform (10 × 3′v2), which left us with three individuals-worth of lung tissue, and seven individuals worth of heart tissue. The data across individuals were then integrated and processed using the Seurat pipeline, resulting in a final dataset consisting of 65,662 cells from the human lung tissue and 238,154 cells from the human heart tissue (26). Pericytes were found annotated based on *CSPG4, PDGFRB*, and *TRPC6* within the Human Lung Cell Atlas, while within the human heart data, pericytes were found annotated based on *RGS5, ABCC9*, and *KCNJ8*. We mapped the annotated pericytes onto the UMAP plot. We compared it with the expression density of the human orthologs of the potential pericyte markers identified in mouse lung and heart.

*CSPG4* or *PDGFRB* was expressed in the human lung and heart (Figures 4A,B; Supplementary Figures 7, 8). We identified high expression of *GUCY1B1, HIGD1B, NDUFAL2, COX4I2*, and *KCNK3* in the annotated pericyte cluster from the Human Lung Cell Atlas. Except for *GUCY1A1, PDE5A, GUCY1B1*, and *DES*, all other markers were non-specific and expressed in other regions. The expression of *HIGD1B, NDUFAL2, COX4I2*, and *KCNK3* was more specific and almost fully overlapped with *CSPG4* or *PDGFRB* and mostly did not overlap with the expression of vascular smooth muscle cell (VSMC) markers *ACTA2, MYH11*, and *TAGLN* (Figure 4A and Supplementary Figure 7). For the human orthologs of the potential pericyte markers identified from the mouse heart tissue, we identified the expression of *NOTCH3* and *HIGD1B* to be relatively high and specific to the annotated pericytes compared with the other markers and relatively did not overlap with the VSMC markers.

### Pericyte Localization in Human Heart Tissue by Spatial Transcriptomic Analysis

**Figure 5:**
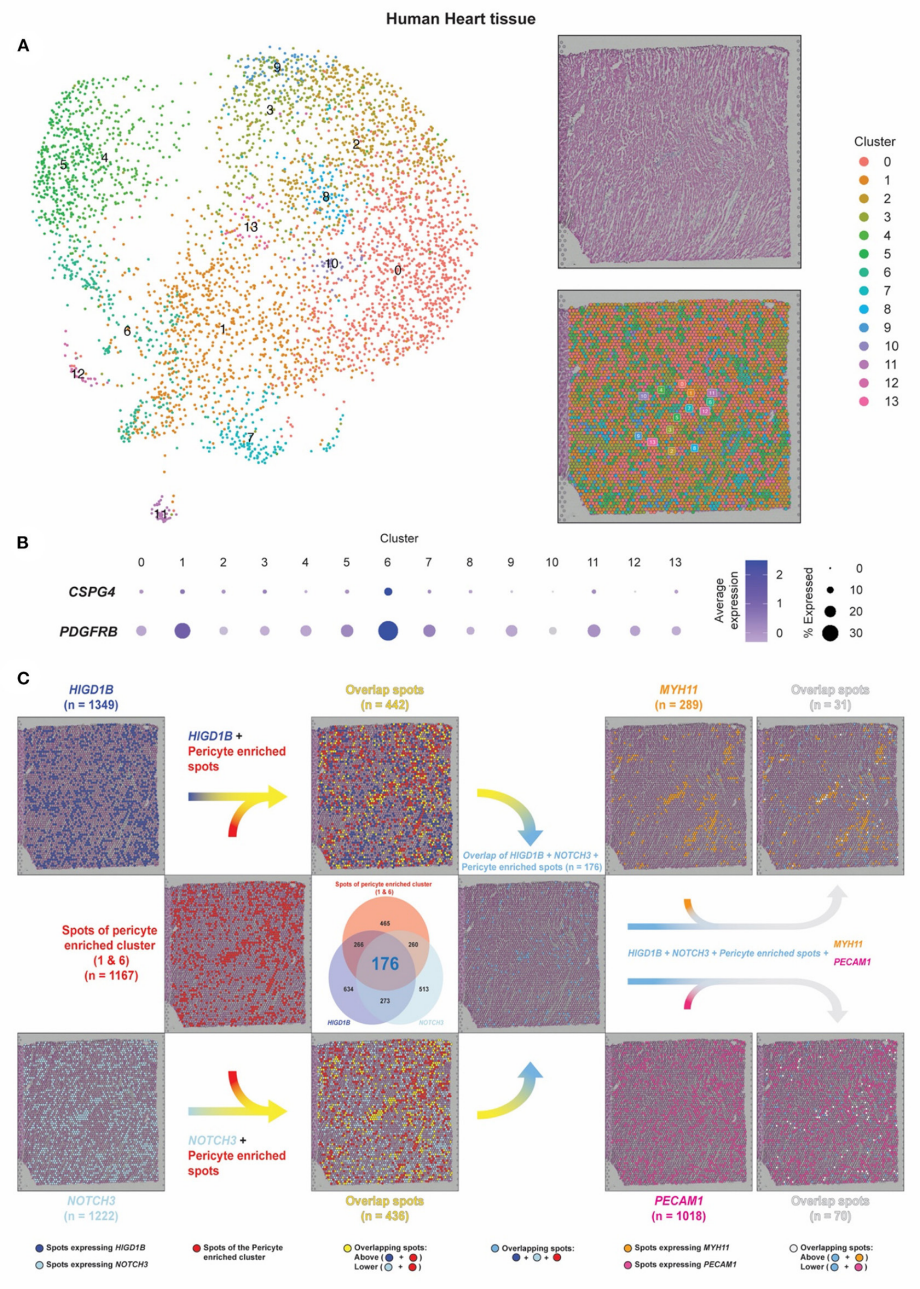
Pericyte transcripts locate on capillaries in human heart tissue by spatial transcriptomic analysis. **(A)** The spatial transcriptomic data of the human heart tissue from the Spatial Gene Expression Dataset by 10 × Genomics Space Ranger (10 × Genomics Space Ranger 1.1.0) was used to investigate the pericyte spatial localization within the heart tissue and compare it with regions of the capillaries and the arteries. **(B,C)** Based on the expression of *CSPG4* and *PDGFRB*, spots of clusters 1/6 were designated as pericyte spots (shown as red dots). Dark blue dots indicate spots that express *HIGD1B*. Cyan dots indicate spots that express *NOTCH3*. Sky blue dots indicate spots that co-express *HIGD1B* and *NOTCH3* among the pericyte spots. Spots that expressed *MYH11* were identified as smooth muscle coverage of arteries (shown as orange dots). Spots that expressed *PECAM1* were identified as endothelial cells and capillary lumens (shown as dark pink dots).

Most scRNA-seq approaches rely on dissociating cells from tissues, thereby losing the crucial spatial context of the cells' locations within the tissue. Techniques such as the image-based single-cell transcriptomics method, multiplexed RNA FISH, hybridized tissue sections to spatially barcoded microarrays, sequencing on intact tissues would simultaneously profile the expression of hundreds or thousands of genes within single cells whose spatial location is preserved (27). We, therefore, investigated the pericyte spatial localization within the human heart tissue. We compared it with regions of capillaries and arteries using the spatial transcriptomic data of human heart tissue from the Spatial Gene Expression Dataset by 10 × Genomics Space Ranger (10 × Genomics Space Ranger 1.1.0) (Figure 5).

Each spot in the dataset represented tissue regions where the transcriptomic reads were obtained. The dataset was projected onto a 2-dimensional map using UMAP, and unsupervised clustering of the spots was performed in which we identified 14 clusters (28, 29). In a sequential tissue section stained by hematoxylin and eosin (H&E), blood cells in dark pink color were visible within the hollow cavities where the same areas as vessels were mainly estimated to reside in. Integrating the transcriptomic and spatial data, the tissue section can be annotated as the same 14 clusters applied to the UMAP plot using the same color-coding (Figure 5A).

Co-expression of *CSPG4* and *PDGFRB* was mainly found in Cluster 1/6, from which 1,167 spots were identified and designated as pericyte spots (Figures 5B,C, red dots). A total of 289 spots that expressed *MYH11* were identified as smooth muscle coverage of arteries (orange dots), while 1,018 spots that expressed *PECAM1* were endothelial cells and capillary lumens (dark pink dots). While capillary spots were randomly distributed, we observed the pattern and shape of arteries as continuous and vessel-like.

We further identified 1,349 spots expressing *HIGD1B* (dark blue dots) and 1,222 spots expressing *NOTCH3* (cyan dots), in which 449 spots co-expressed both *HIGD1B* and *NOTCH3* (Venn diagram, 176+273) (Figure 5C). After colocalization with Cluster 1/6, 37.87% overlapped spots were co-expressing *HIGD1B* (yellow dots on the upper-side flow), while 37.36% overlapped spots were co-expressing *NOTCH3* (yellow dots on the lower-sided flow). A total of 176 dots (6.8%) co-expressed *HIGD1B* and *NOTCH3* among the pericyte spots (sky blue dots on the middle right-sided flow), in concordance with their expected location independent from *MYH11* and *PECAM-1*. Although these spots were not cells (but rather aggregates of transcripts) and would have lower resolution than immunofluorescence staining, our spatial data revealed that pericytes were exclusively located on distal vessels, not on large vessels. This method also opened a new avenue to evaluate quantitative and spatially resolved maps of gene expression at a transcriptomic level no longer dependent on the accessibility of their corresponding antibodies.

## Discussion

Despite their functional importance and implications being one of the major components in capillary networks and indispensable in capillary homeostasis and function throughout the body in all organs, pericytes remain poorly characterized. Pericytes are also identified to play vital roles in vessel stabilization (30, 31), blood brain barrier formation (32), immune cell guidance (33), blood flow regulation (34), tissue repair, (9) and pathological scarring/fibrosis (35, 36). A significant challenge to studying pericytes is the lack of a consensus definition or cellular marker that can distinguish them from other mural cell populations. Additionally, genes and their enriched pathways that govern pericyte unique function in an organ-specific manner have not been demonstrated, primarily due to insufficient methodologies that can investigate a single pericyte cell type from various tissues in parallel.

With the advent of scRNA-seq technologies, our study investigated the transcriptomic landscape of pericytes that may be common to or differ across four tissues. Differentially expressed genes from *Cspg4*/*Pdgfrb* expressing compared to non-expressing cells revealed 18 lung-specific, 4 heart-specific, 22 kidney-specific, and 4 bladder-specific pericyte markers (Figure 2). The Venn diagram also suggested the commonly expressed pericyte transcriptome across four organs. *Gucy1a3*, the main receptor for nitric oxide, *Ndufa4l2*, which mediates activation of oxidative phosphorylation and produces ROS in the mitochondria; and *Myl9*, which regulates muscle contraction by modulating the ATPase activity of myosin heads, are all identified as potential pericyte markers in both the lung and kidney (37–39). Recently, *NDUFA4L2*, the human ortholog of *Ndufa4l2*, is involved in the vascular remodeling of smooth muscles in hypoxic pulmonary arterial hypertension (40). Although *Myl9* is well-expressed in muscles, it is also expressed in pericytes of the murine cortex (41). *Gm13889* and *Notch3* were identified as potential pericyte markers in the heart and kidney. Although not much is known about *Gm13889, Notch3* is involved in neural development and brain pericyte proliferation (42). *Higd1B*, which belongs to the hypoxia-inducible gene 1 (HIG1) domain family and is involved in cell survival by maintaining mitochondrial integrity under hypoxia conditions, was identified as a potential pericyte marker in both the lung and heart (43). *Cox4i2*, which catalyzes the electron transfer from reduced cytochrome c to oxygen, is identified as a potential pericyte marker in the lung, kidney, and bladder (44). Human *COX4I2* is differentially expressed in pericytes and annotated as a pericyte marker in the Human Lung Cell Atlas (24). Our study also identified tissue-specific pericyte markers, which may potentially serve as promising targets for organ-specific delivery of therapeutics (e.g., AAV) that process efficient target specificity and reduce the risk of side effects. Based on the differentially expressed genes, we conducted a GSEA analysis to identify activated pathways in pericytes. Intriguingly, vascular smooth muscle contraction, dilated cardiomyopathy, and the focal adhesion pathways were suggested as commonly activated among all four tissues (Figure 3).

It was vital to ensure the results from mouse datasets could be translatable to human pericytes. Thus, the identified pericyte markers in mouse lung and heart were compared with the human lung and heart scRNA seq datasets. Overall, we found considerable overlapped gene expression between mouse and human tissues. Human orthologs of *Ndufal2, Cox4i2, Kcnk3*, and *Higd1b* were highly relevant and specific to the annotated lung pericyte clusters, while the human ortholog of *Notch3* and *Higd1b* was found in the heart pericyte cluster. Lastly, applying gene expression into context and delineating the spatial arrangement of cell types, we visualized the pericyte/EC/SMC distribution and further presented *Notch3* and *Higd1b* as pericyte markers and their spatial locations on a human heart tissue (Figure 5).

A significant challenge for studying pericyte pathobiology was lacking a consensus definition that helps distinguish pericytes from other mural cell populations, including smooth muscle cells, fibroblasts, myofibroblasts, and others. However, single-cell analysis of brain vasculature, particularly the distinguishment for brain pericytes and other mural cells, has been well-investigated (45–47). Murine brain pericytes share markers, including *Vtn, Higd1b, S1pr3, Mcam, Ifitm1, Baiap3*, and *Ehd3* with lung pericytes. Intriguingly, brain pericytes have substantial organotypic differences on markers such as *Anpep*, which is not expressed by lung pericytes (45). One of the top commonly altered pathways in all organ pericytes was associated with vascular smooth muscle contraction in our pathway analysis. This result was consistent with our most recent work that suggested NG2-expressing pericytes became smooth muscle like under hypoxia-induced pulmonary hypertension (48). Additionally, heart pericytes may serve as a progenitor for smooth muscle cells during embryonic heart development. Another lineage analysis suggests that kidney pericytes may differentiate into most smooth muscle actin expressing myofibroblasts during fibrosis (49). In the Human Lung Cell Atlas project, lung pericytes are identified using *COX4I2, TBX5*, and *KCNK3* and its potential implication in pulmonary hypertension is proposed (24). These abovementioned studies laid some groundwork to elucidate the identification and mechanisms of pericytes; however, their refined biological role and potential for drug targeting still need more investigation.

Our work had several limitations. Only four datasets were included in our analysis due to minimum *Cspg4* and *Pdgfrb* expressions across all other organs. Therefore, larger sample sizes were needed to corroborate these results. These results should be further validated using the single-cell data generated by the FACS method. Our study did not include brain pericytes due to a lack of Droplet data on brain tissues. We also did not consider age as one of the variable factors. Pericytes may have different features that may be unique across developmental processes. Therefore, to identify diverse pericytes across developmental and differentiation processes, we needed to specify the pericyte population in each age group. Additionally, we did not look for variance between men and women, which would provide us with more insights into potential sex differences in pericyte biology and cardiovascular disease risk. There may be different pericyte subpopulations among the same organ between mice and humans regarding its heterogeneity. This subtype variation will be reflected at the transcriptomic level and needed more extensive analysis of co-expression of both pericyte markers and other mural cell markers. For instance, after pericytes were identified using *Pdgfrb* and *Cspg4*, sub-clustering these double-positive cells would provide a clearer understanding of subpopulation signature genes.

While the identified genes tested against the human lung and heart single-cell datasets have a high potential as pericyte unique markers, additional protein analysis and functional validation will be required to confirm each pericyte phenotype's putative role using the antibody RNAScope staining. When going through the methods of several scRNAseq experiments used in this study, we noted that lung tissues underwent enzymatic digestion to achieve single-cell suspension before subjecting to scRNAseq analysis in the traditional dissociation method. The percentage of cells annotated as pericyte clusters seemed to be relatively small compared with the total cell population in most publicly available datasets. It was also possible that during dissociation of the tissues or FACS-based method, pericytes were not completely separated from endothelial cells, thus forming doublets and being excluded from the analysis. An optimized and enriched pericyte isolation method to preserve viability and subsequently pericyte scRNA-seq atlas will be valuable in addressing their unknown nature as indicated above. Lastly, pericyte spatial orientation alongside the vascular tree structure requires validation (for instance, by spatial scRNA-seq methods or Nanostring GeoMax) and their resulting data will help us to understand pericyte distribution or morphological change in the pathogenesis of diseases, especially clinical meaningful if we can identify them on patient biopsy samples during disease progression.

Our study provides important new insights into organ-specific pericyte localization using single-cell RNAseq from mouse and human databases and evaluating pericyte spatial transcriptomic orientation on human heart tissues. These datasets are effective in identifying tissue-specific markers and in understanding biological processes and active pathways in other diverse cell types (16, 24, 25). The single cell and spatial data revealed that pericyte transcripts were exclusively located on distal vessels, which was consistent with pericyte immunostaining results (1, 48). This method also revealed the relationship between single cell and their spatial transcriptomics data and thus could spatially map any cell types of interest across the tissue.

In conclusion, our work provides innovative and insightful knowledge on facilitating the identification of pericyte phenotypes across different tissues. Future studies with a wider range of tissues considering developmental processes and focusing on the trajectory of pericyte differentiation will provide us with unique markers for organ-specific pericytes, which would aid us in understanding and characterizing the role of pericyte plays in diverse biological processes and pathogenesis of related diseases.

## Methods

### Data Availability

In this manuscript, we analyzed microfluidic droplet based scRNA-seq data processed using the 10 × Genomics platform (10 × 3′v2) of the (1) Tabula Muris Senis, (2) Human Lung Cell Atlas, and the (3) Cells of the human heart that were all obtained from the Cellxgene collections (https://cellxgene.cziscience.com) (16, 24, 25). The specific pre-processed Seurat objects for these datasets we obtained are as follows:

1. The lung, heart, kidney, and bladder tissue of the Tabula Muris Senis dataset: (https://cellxgene.cziscience.com/collections/0b9d8a04-bb9d-44da-aa27-705bb65b54eb).
2. The Human Lung Cell Atlas dataset: (https://cellxgene.cziscience.com/collections/5d445965-6f1a-4b68-ba3a-b8f765155d3a).
3. The dataset of the cells of the adult human heart: (https://cellxgene.cziscience.com/collections/b52eb423-5d0d-4645-b217-e1c6d38b2e72).

In addition, spatial transcriptomics for the human heart was obtained from the Spatial Gene Expression Dataset by 10x Genomics Space Ranger (10x Genomics Space Ranger 1.1.0) at: (https://www.10xgenomics.com/resources/datasets/human-heart-1-standard-1-1-0).

### Pre-processing Single-Cell RNA Sequencing Data

The obtained scRNA-seq data were analyzed based on the pipeline of the Seurat R package (26). The dataset of each subject and tissue was identified and processed through the Seurat pipeline in which the feature counts were log-normalized and subsequently scaled. The top 2,000 variable features were then selected based on the Variance Stabilizing Transformation method for integration (50). Within each tissue, the datasets of the according subjects were then integrated using the IntegrateData function of Seurat. The annotated cell types within the Seurat objects of the human lung dataset and the human heart datasets were used to identify pericytes.

### Dimensionality Reduction and Clustering

For each integrated scRNA-seq dataset, principal component (PC) analysis was conducted on the highly variable genes to reduce the dimensionality. Based on the percentage of variance explained by the top PCs, we selected the top 45 PCs for the mouse kidney, the top 30 PCs for the mouse bladder, and the top 35 PCs for the remaining (lung and heart of both the mouse and human) datasets to conduct the downstream analysis. Based on the selected PCs, for each dataset, cells were projected into a 2-dimensional space using the Uniform Manifold Approximation and Projection (UMAP) algorithm (28). We conducted an unsupervised clustering of single cells using the FindCluster function in Seurat, setting the resolution measure to 0.5, which constructs a shared nearest neighbor graph by identifying the *k*-nearest neighbors for each cell in the PC space and weights the edges between the cells based on the number of the *k*-nearest neighbors or their Jaccard similarity (29). The densely connected cells are then considered as clusters in which the modularity is optimized using the Louvain community detection method (26, 29).

### Differential Gene Expression Analysis

Differential expression analysis was conducted by comparing the expression of stringent pericytes (cells that co-expressed *Cspg4* and *Pdgfrb* within the identified pericyte-enriched cluster) with all other cells according to the tissue for the Tabula Muris Senis dataset using a Wilcoxon rank sum test (26). Genes with an adjusted *p*-value below 0.05 that are expressed in more than 80% of the stringent pericytes and in <5% of all other cells were selected as potential pericyte markers according to the tissue.

### Gene Set Enrichment Analysis

The Gene Set Enrichment Analysis (GSEA) (17) was used to interpret the gene expression data using the software available at https://www.gsea-msigdb.org/gsea/index.jsp. A pre-ranked GSEA determines whether genes within an *a priori* defined specific gene tend to occur toward the top or the bottom of a pre-ranked gene list, which then identifies whether the corresponding gene set shows statistically significant differences between two biological states. We generated pre-ranked gene lists by sorting the DE genes based on their log2 fold change from the DE analysis in a descending order. GSEA was conducted using the resulting pre-ranked gene lists and compared with the gene sets defined by the Kyoto Encyclopedia of Genes and Genomes (KEGG) pathway gene sets. A permutation test estimates the statistical significance of the enrichment score. GSEA generates a version of the dataset with phenotype labels randomly scrambled, produces the corresponding ranked list, and then recomputes the enrichment score of the gene set for this permuted dataset. Pathways with an adjusted *q*-value below 0.05 in this analysis were considered statistically significant.

## Data Availability Statement

The data presented in the study are deposited in the the Cellxgene collections (https://cellxgene.cziscience.com). The lung, heart, kidney, and bladder tissue of the Tabula Muris Senis dataset: (https://cellxgene.cziscience.com/collections/0b9d8a04-bb9d-44da-aa27-705bb65b54eb). The Human Lung Cell Atlas dataset: (https://cellxgene.cziscience.com/collections/5d445965-6f1a-4b68-ba3a-b8f765155d3a). The dataset of the cells of the adult human heart: (https://cellxgene.cziscience.com/collections/b52eb423-5d0d-4645-b217-e1c6d38b2e72).

## Ethics Statement

The study was approved by the Institutional Biosafety Committee of Boston Children's Hospital.

## Author Contributions

S-HB and KY planned and performed experiments. S-HB was responsible for data analysis. S-HB, EM, HK, KG, BR, and KY provided intellectual input. BR and KY conceived the study. S-HB, BR, and KY wrote the manuscript. The order of the authors was decided to depend on the combined contribution of intellectual input and experiments performed. All authors contributed to the article and approved the submitted version.

## Funding

This work was supported by NIH/NHLBI 5R01HL150106-02, ATS/PHA Aldrighetti Research Award for Young Investigators and Pulmonary Hypertension Accelerated Bayer Awards (to KY).

## Conflict of Interest

The authors declare that the research was conducted in the absence of any commercial or financial relationships that could be construed as a potential conflict of interest.

## Publisher's Note

All claims expressed in this article are solely those of the authors and do not necessarily represent those of their affiliated organizations, or those of the publisher, the editors and the reviewers. Any product that may be evaluated in this article, or claim that may be made by its manufacturer, is not guaranteed or endorsed by the publisher.

## Supplementary Material

Supplementary Figure 1 UMAP plots depict expressions of *Cspg4* and *Pdgfrb* across different organs of the Tabula Muris Senis dataset. UMAP showing expression levels of *Cspg4* (top) and *Pdgfrb* (bottom) across organs of the Tabula Muris Senis dataset.

Supplementary Figure 2 UMAP plots depict expressions of *Cspg4, Pdgfrb*, and vascular smooth muscle cell (VSMC) markers in the mouse lung. UMAP plots showed expression levels of *Cspg4, Pdgfrb*, and VSMC markers (*Acta2, Myh11, Tagln*) from the Tabula Muris Senis lung droplet dataset.

Supplementary Figure 3 UMAP plots depict expression of *Cspg4, Pdgfrb*, and vascular smooth muscle cell (VSMC) markers in the mouse heart. UMAP plots showed expression levels of *Cspg4, Pdgfrb*, and VSMC markers (*Acta2, Myh11, Tagln*) from the Tabula Muris Senis heart droplet dataset.

Supplementary Figure 4 UMAP plots depict expression of *Cspg4, Pdgfrb*, and vascular smooth muscle cell (VSMC) markers in mouse kidney. UMAP plots showed expression levels of *Cspg4, Pdgfrb*, and VSMC markers (*Acta2, Myh11, Tagln*) from the Tabula Muris Senis kidney droplet dataset.

Supplementary Figure 5 UMAP plots depict expression of *Cspg4, Pdgfrb*, and vascular smooth muscle cell (VSMC) markers in the mouse bladder. UMAP plots showed expression levels of *Cspg4, Pdgfrb*, and VSMC markers (*Acta2, Myh11, Tagln*) from the Tabula Muris Senis bladder droplet dataset.

Supplementary Figure 6 Heatmap plot depicts expression levels of potential pericyte markers. Heatmap plot showed expression levels of identified tissue-specific pericyte markers from four tissues.

Supplementary Figure 7 Expression levels of murine pericyte markers are identified in the human lung single cell RNAseq dataset. Density plot of the expression of human orthologs of potential pericyte markers identified from the mouse lung were further identified on the collection of the single cells of the Human Lung Cell Atlas.

Supplementary Figure 8 Expression levels of murine pericyte markers are identified in the human heart single-cell RNAseq dataset. Density plot of the expression of human orthologs of potential pericyte markers identified from the mouse heart were further identified on the collection of the single cells of the adult human heart.
