# Supplementary figures and images for: Single Cell Transcriptomic Analysis Reveals Organ Specific Pericyte Markers and Identities
Source: Front Cardiovasc Med. 2022 Jun 1;9:876591. doi: 10.3389/fcvm.2022.876591 (PMC9199463; doi:10.3389/fcvm.2022.876591)

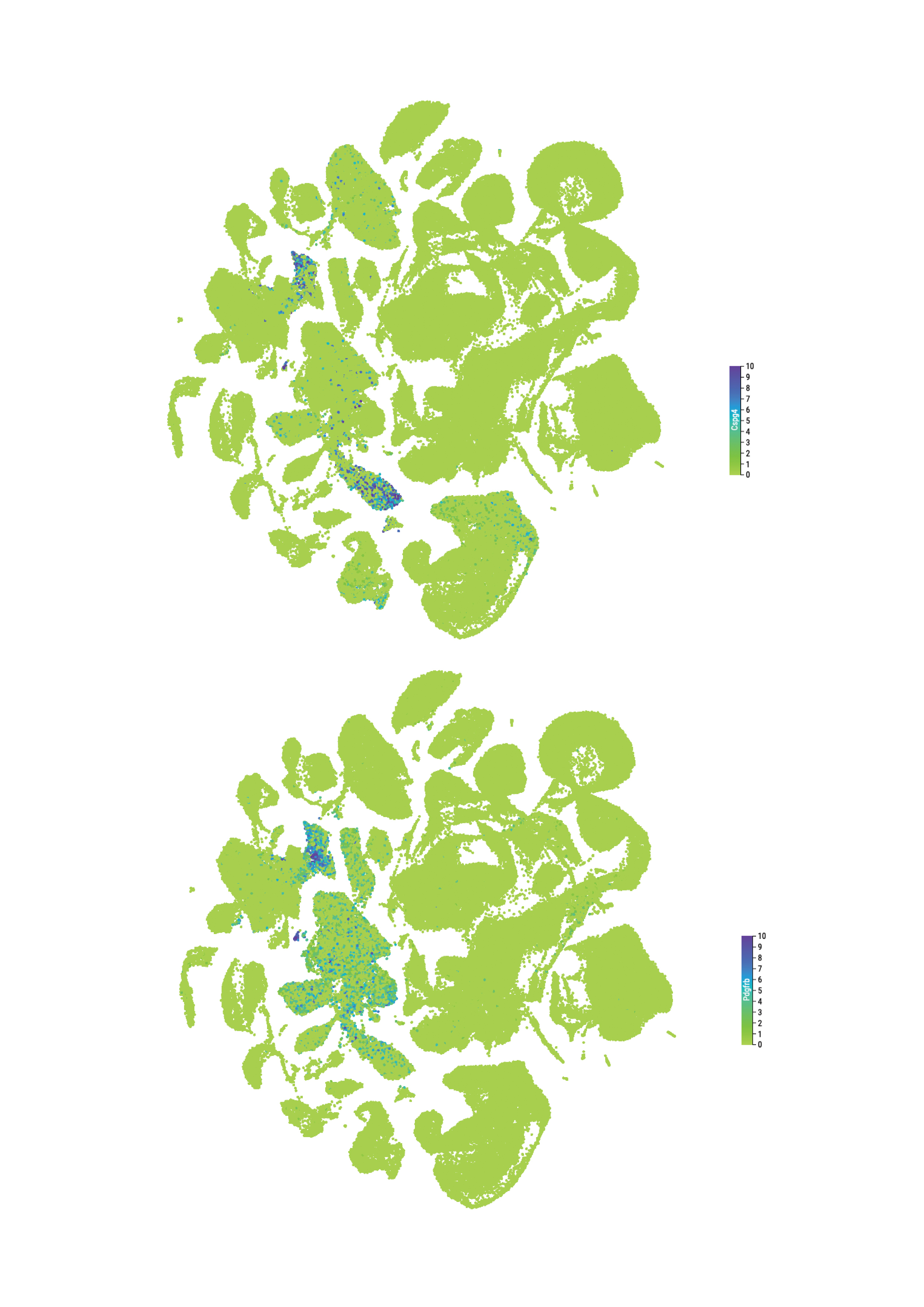

Supplement: Supplementary Figure 1 — UMAP plots depict expressions of Cspg4 and Pdgfrb across different organs of the Tabula Muris Senis dataset. UMAP showing expression levels of Cspg4 (top) and Pdgfrb (bottom) across organs of the Tabula Muris Senis dataset. [file Image_1.JPEG]

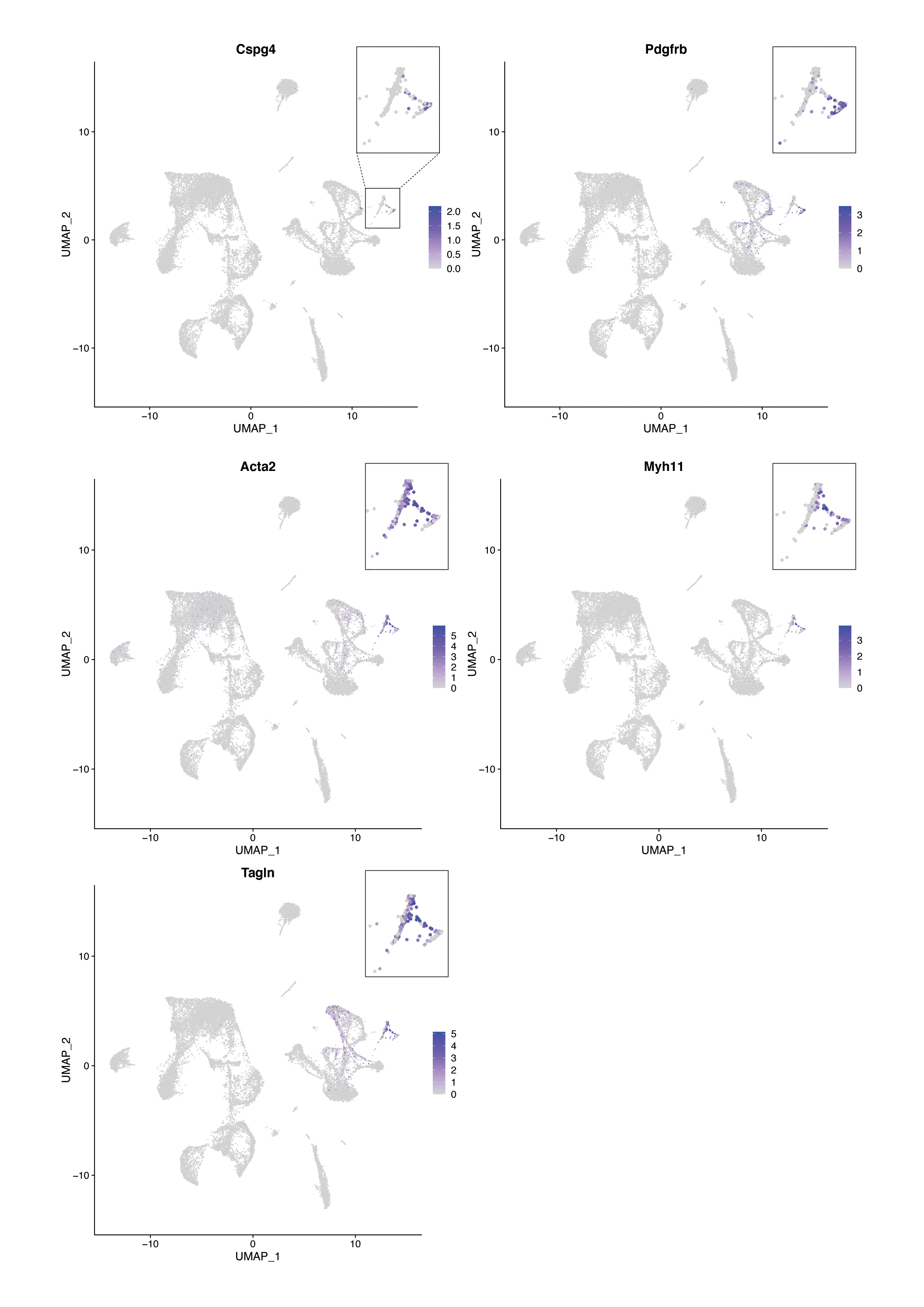

Supplement: Supplementary Figure 2 — UMAP plots depict expressions of Cspg4, Pdgfrb, and vascular smooth muscle cell (VSMC) markers in the mouse lung. UMAP plots showed expression levels of Cspg4, Pdgfrb, and VSMC markers (Acta2, Myh11, Tagln) from the Tabula Muris Senis lung droplet dataset. [file Image_2.JPEG]

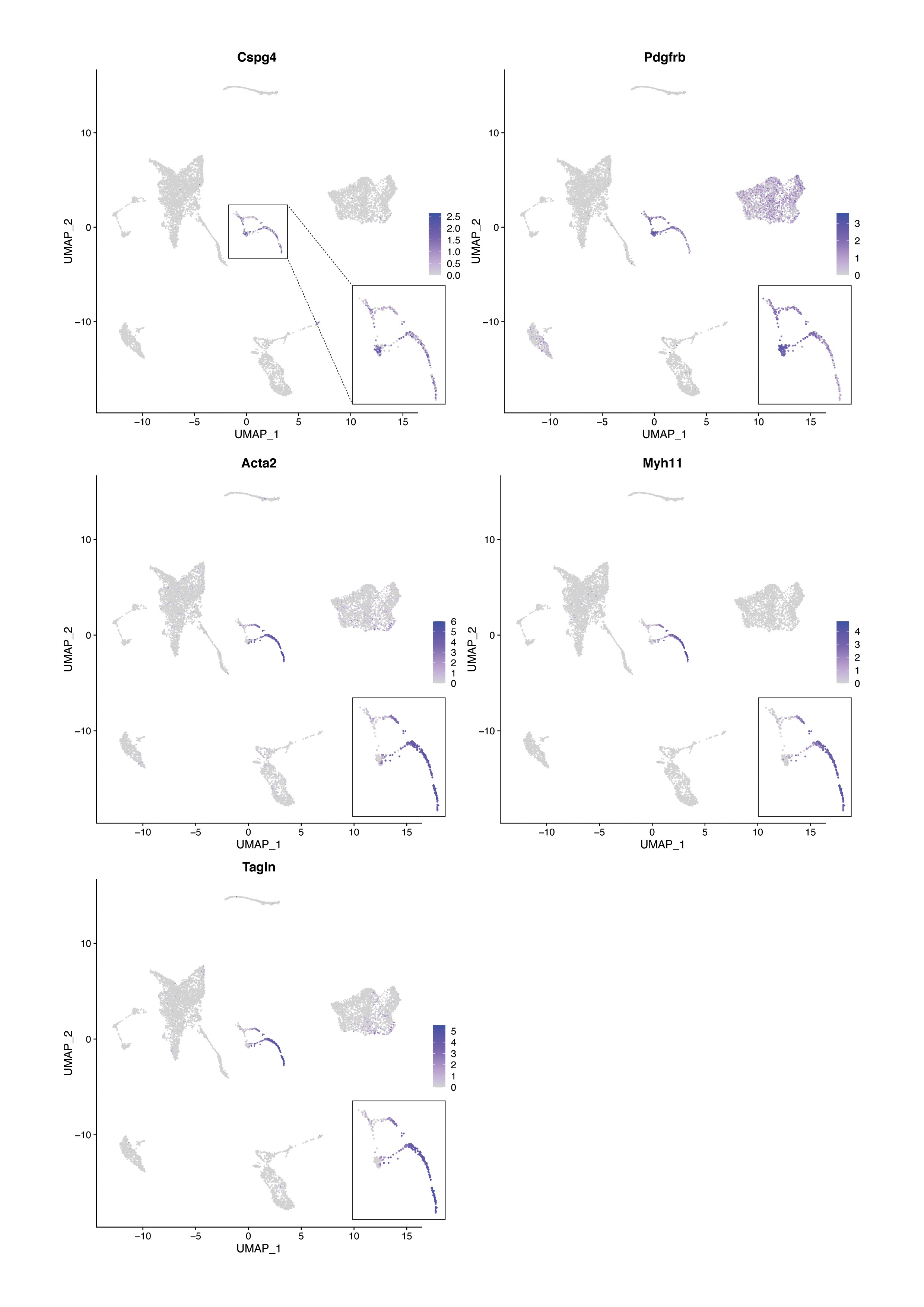

Supplement: Supplementary Figure 3 — UMAP plots depict expression of Cspg4, Pdgfrb, and vascular smooth muscle cell (VSMC) markers in the mouse heart. UMAP plots showed expression levels of Cspg4, Pdgfrb, and VSMC markers (Acta2, Myh11, Tagln) from the Tabula Muris Senis heart droplet dataset. [file Image_3.JPEG]

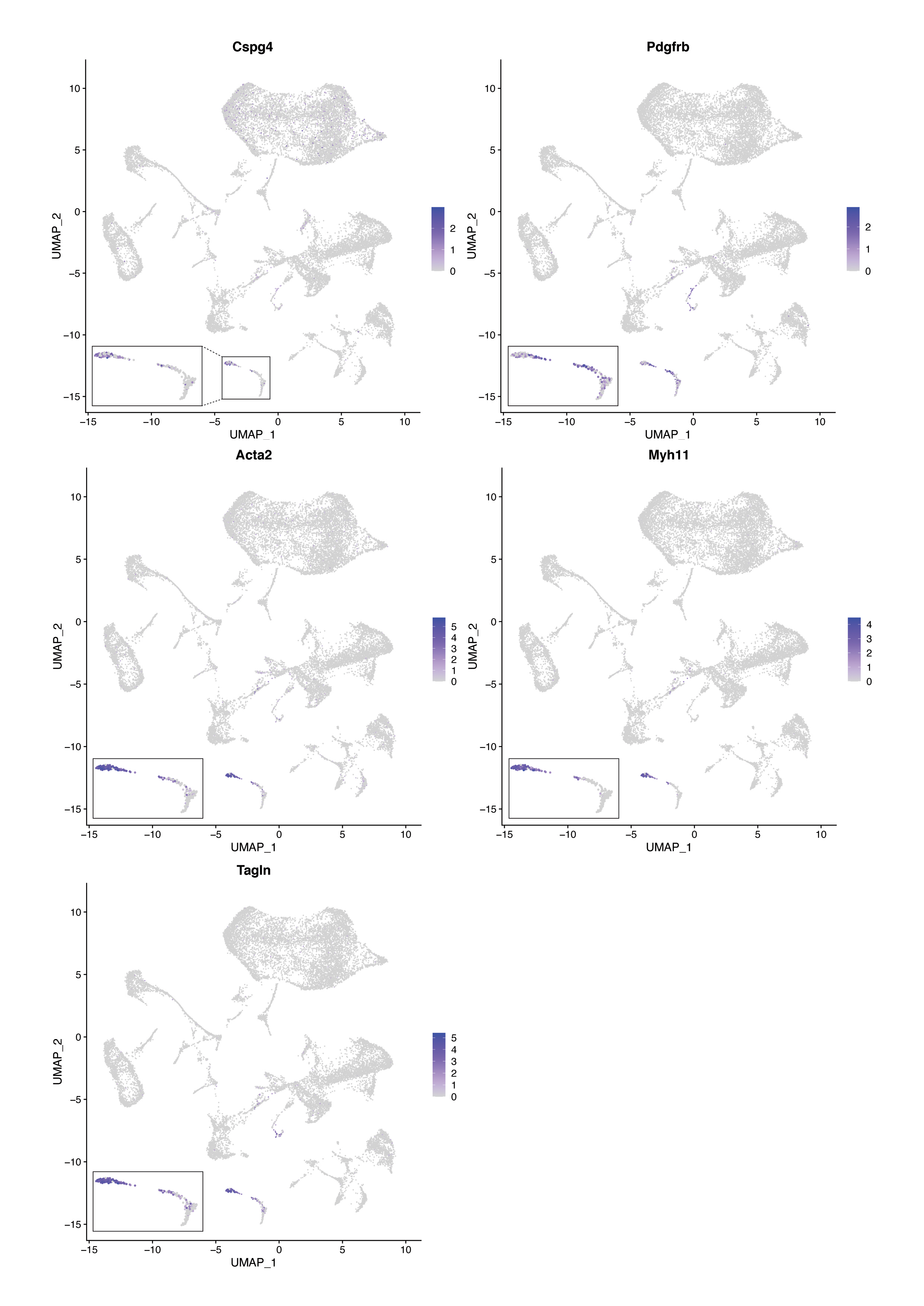

Supplement: Supplementary Figure 4 — UMAP plots depict expression of Cspg4, Pdgfrb, and vascular smooth muscle cell (VSMC) markers in mouse kidney. UMAP plots showed expression levels of Cspg4, Pdgfrb, and VSMC markers (Acta2, Myh11, Tagln) from the Tabula Muris Senis kidney droplet dataset. [file Image_4.JPEG]

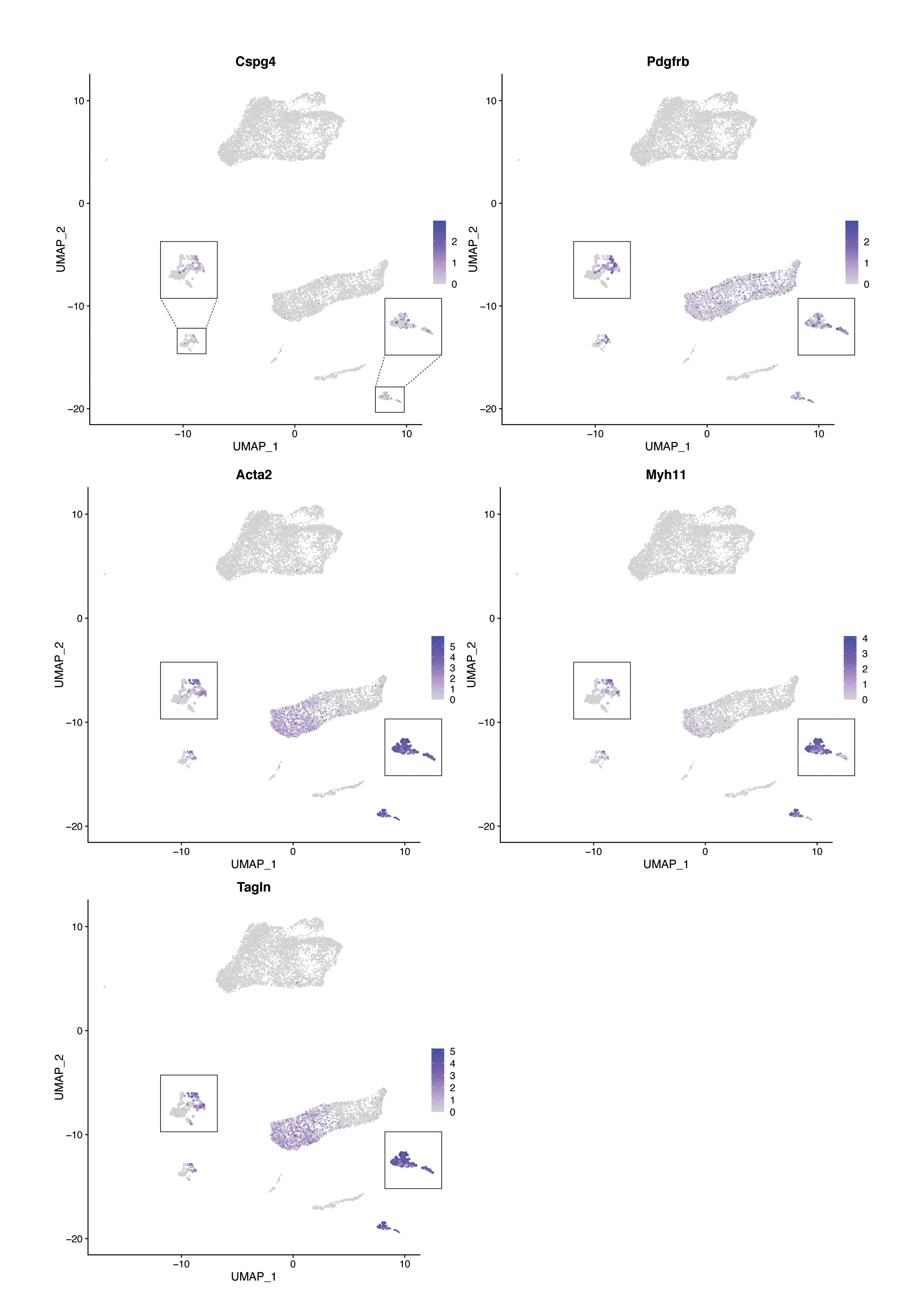

Supplement: Supplementary Figure 5 — UMAP plots depict expression of Cspg4, Pdgfrb, and vascular smooth muscle cell (VSMC) markers in the mouse bladder. UMAP plots showed expression levels of Cspg4, Pdgfrb, and VSMC markers (Acta2, Myh11, Tagln) from the Tabula Muris Senis bladder droplet dataset. [file Image_5.JPEG]

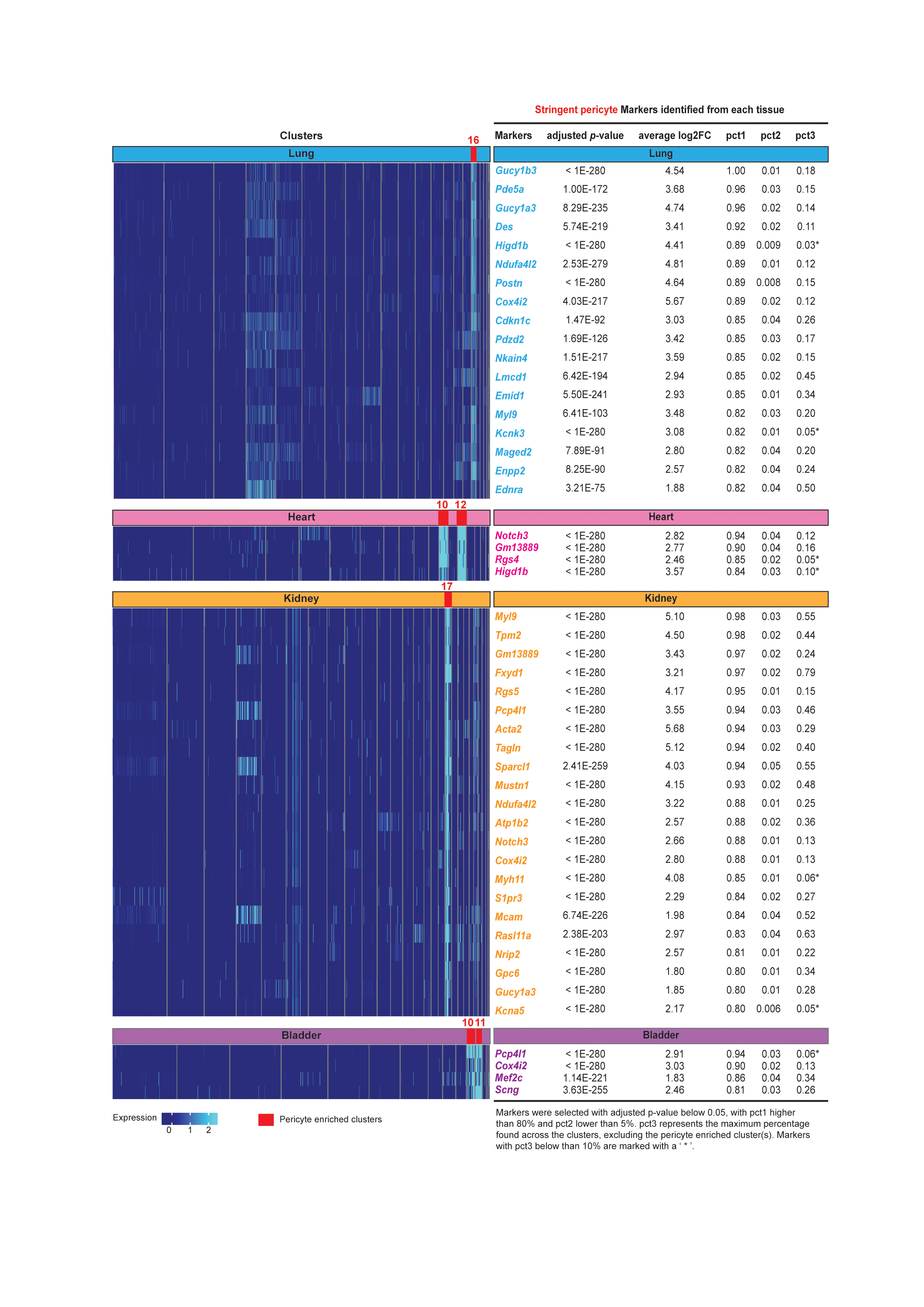

Supplement: Supplementary Figure 6 — Heatmap plot depicts expression levels of potential pericyte markers. Heatmap plot showed expression levels of identified tissue-specific pericyte markers from four tissues. [file Image_6.JPEG]

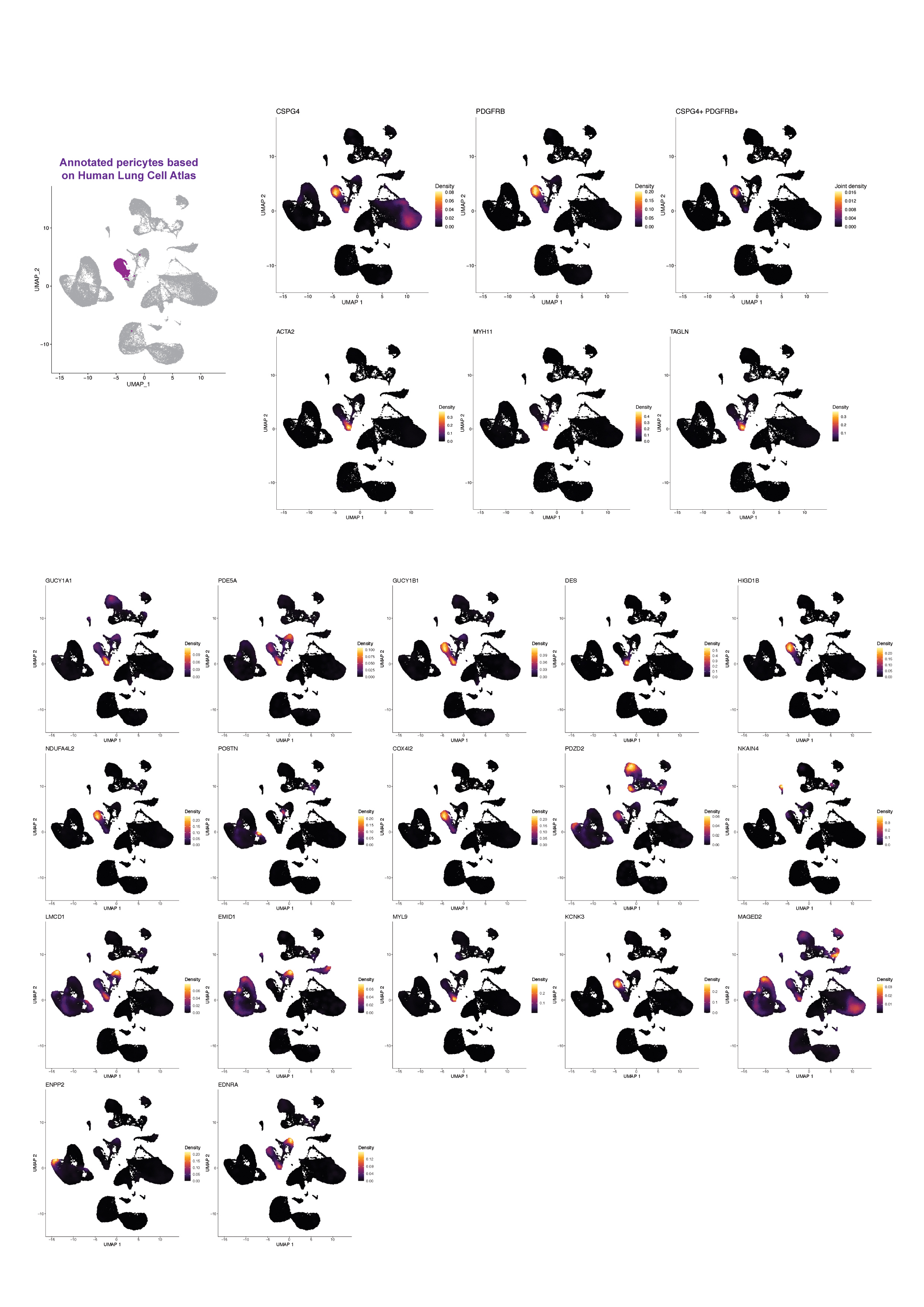

Supplement: Supplementary Figure 7 — Expression levels of murine pericyte markers are identified in the human lung single cell RNAseq dataset. Density plot of the expression of human orthologs of potential pericyte markers identified from the mouse lung were further identified on the collection of the single cells of the Human Lung Cell Atlas. [file Image_7.JPEG]

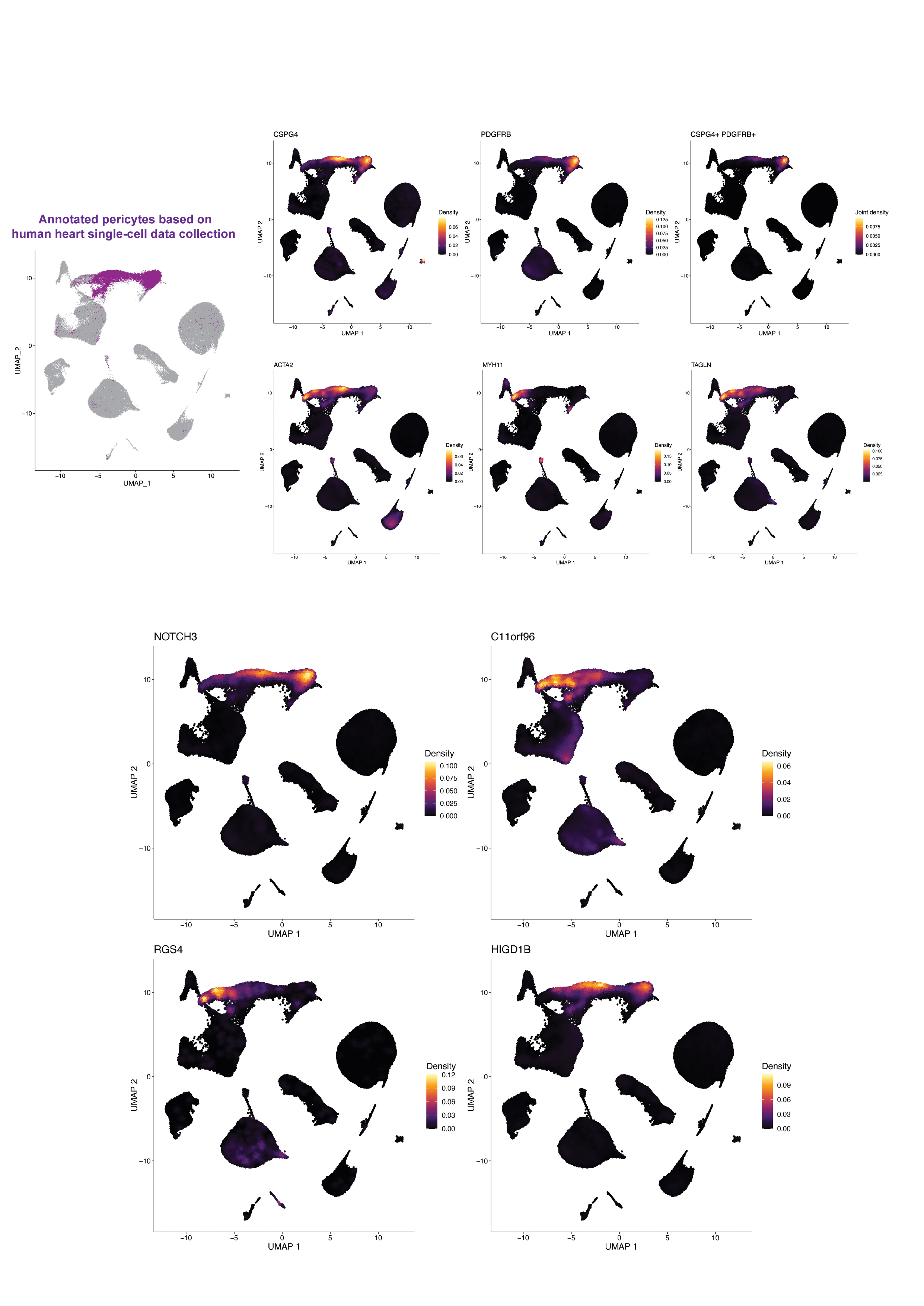

Supplement: Supplementary Figure 8 — Expression levels of murine pericyte markers are identified in the human heart single-cell RNAseq dataset. Density plot of the expression of human orthologs of potential pericyte markers identified from the mouse heart were further identified on the collection of the single cells of the adult human heart. [file Image_8.JPEG]
